# Outbreak of novel coronavirus infection (COVID-19) in a child and adolescent psychiatric ward: Characteristics and responses

**DOI:** 10.20407/fmj.2023-003

**Published:** 2023-08-28

**Authors:** Koichi Furuhashi

**Affiliations:** 1 Department of Child and Adolescent Psychiatry, National Hospital Organization Higashiowari National Hospital, Nagoya, Aichi, Japan; 2 Department of Psychiatry, School of Medicine, Fujita Health University, Toyoake, Aichi, Japan

**Keywords:** Coronavirus Infectious Disease (COVID-19), Child and Adolescent Psychiatric Ward, Inpatient, Post-Traumatic Stress Disorder, Autism Spectrum Disorder

## Abstract

**Background:**

The novel coronavirus disease (COVID-19) pandemic has no end in sight. Currently, the emphasis is on policies aimed at easing movement restrictions and maintaining socio-economic activities. However, infection control in psychiatric hospitals has been challenging. There have been reports on the impact on mental health and outpatient/inpatient treatment environments in the field of child psychiatry. An outbreak of COVID-19 was experienced in a child and adolescent psychiatric ward, and considering that there have been few similar reports, it was deemed meaningful to accumulate such experiences.

**Case presentation:**

Three COVID-19-positive cases, all 14-year-old girls, were confirmed in a cluster among seven hospitalized patients in a child and adolescent psychiatric ward. Two patients presented symptoms of upper respiratory inflammation and one was asymptomatic. The main psychiatric diagnoses were post-traumatic stress disorder in one patient and autism spectrum disorder in the other two patients. The entire hospital ward was designated as a red zone (contaminated area), and infection control measures were adopted, such as halting group activities, wearing masks, and maintaining distance between patients. Additionally, it was necessary to use the infection control ward as it was difficult to ensure patient compliance.

**Conclusion:**

Infection control in COVID-19 clusters at child and adolescent psychiatric wards is difficult due to patient characteristics and symptoms. Restricted activities and care also result in psychobehavioral consequences, regardless of infection status. To achieve both infection control and a better treatment environment, it is necessary to make careful preparations while learning from these experiences.

## Introduction

The novel coronavirus disease (COVID-19) has spread rapidly worldwide since 2019. Owing to the emergence of new mutant strains, waves of infection have continued and there seems to be no end in sight. Under such circumstances, there has been advocacy of policies to maintain socioeconomic activities while easing restrictions on movement. However, once the infection spreads in medical institutions, normal medical care becomes difficult to maintain. Particularly, several reports elucidated the difficulties involved in responses at psychiatric hospitals. Individuals with mental disorders have a high proportion of physical comorbidities such as obesity, hypertension, and metabolic syndrome, and they may be more susceptible to COVID-19 infection and are more likely to experience a more severe COVID-19 infection.^1^ In the hospital environment, which usually features closed wards, it is difficult to ensure social distancing, install disinfectants (owing to a risk of accidental ingestion), and thoroughly ensure hygiene (such as wearing masks). Insufficient staff training and skills and weak organizational and community support have also been pointed out as factors affecting COVID-19 treatment.^1,2^ Medical resources for treating COVID-19 are limited within psychiatric hospitals, and general hospitals that undertake the treatment of infected patients with mental illness have difficulties accepting patients.^3^ For these reasons, strict infection control measures are required.

In the field of child psychiatry, there have been reports on the impact of the COVID-19 pandemic on children’s mental health and the treatment environment for hospitalized patients and outpatients. For example, it has been pointed out that there have been increases in anxiety and depressive symptoms, suicidal ideation, self-harm, and suicide and that children with neurodevelopmental disorders and other neurodiverse conditions have higher psychological distress and greater behavioral problems.^4,5^ The clinical effects have resulted in a decrease in the number of hospitalized patients and outpatients, restrictions on hospitalized patients’ ability to go out or spend the night elsewhere, suspension of group activities, and restrictions on behavior during hospitalization, making it difficult to adaptively release impulsiveness and aggression. Consequently, the number of troubles in wards increased, the relative severity of hospitalized patients’ symptoms increased, and the duration of hospitalization lengthened.^6^ Additionally, an increase in the number of emergency hospitalizations in child and adolescent psychiatric wards, including increases in the percentage of girls and suicidal tendencies, have been reported.^7^ Moreover, COVID-19-related stressors reportedly contributed to the majority of patients being admitted to child and adolescent psychiatric wards.^8^

The onset of COVID-19 was experienced in multiple patients in a child and adolescent psychiatric ward. As it was the first time inpatients were infected at the specific hospital, the advice of the Infection Control Committee was followed and there was responded with caution. Tilmanne et al. reported on a case of a COVID-19 outbreak in a child and adolescent psychiatric ward where strict infection control measures were able to control the spread of infection.^9^ Krass et al. also reported on a COVID-19 outbreak at a psychiatric hospital, indicating that COVID-19-positive child and adolescent patients were gathered into a pediatric COVID-19 ward, with 20%–30% experiencing symptoms such as nausea/vomiting, coughing, sore throat, and shortness of breath. However, none required oxygen administration or transfer to other hospitals for treatment. Additionally, there was an increase in depressive and anxiety symptoms during the primary stage.^10^ Furthermore, according to Israeli et al., the outbreak of COVID-19 in a child and adolescent psychiatric ward also affected psychiatric treatment due to restrictions on contact with the outside world, such as restrictions on patients’ ability to go out or spend the night elsewhere. Most patients who tested positive had mild or no symptoms. Although the unit was divided into COVID-19-positive and negative patients, the main difficulty experienced by staff was taking measures against infection. Despite this, the patients’ clinical statuses were stable. No psychobehavioral problems or exacerbation of suicide-related symptoms were observed.^11^

COVID-19 is a new infectious disease, and the elucidation of its pathology and the development of treatment methods are progressing daily. Additionally, there are differences in COVID-19-related social and legal regulations as well as treatment resources at child and adolescent psychiatric wards depending on the country or region. As previously mentioned, only a few cases of responses to COVID-19 outbreaks in child and adolescent psychiatric wards have been reported. Therefore, the presented report of experience below is considered to be significant.

## Cluster progress and infection control

The hospital where this study was conducted is a specialized psychiatric hospital, which includes a child and adolescent psychiatric ward with 14 beds, consisting of four private rooms, two four-bed rooms, and two protection rooms. During the follow-up period from April to May 2022, seven patients (aged 13–15 years, all girls) were hospitalized. The main psychiatric diagnosis was post-traumatic stress disorder in five patients and autism spectrum disorder in two patients. All seven patients had a history of intervention at a child guidance center due to abuse from their caregivers, and three patients tested positive for COVID-19 during the observation period. The source of infection was presumed to be a staff member (nurse). In the following case reports, the day the employee tested positive was defined as Day 0 (because they had a fever at home, visited a nearby doctor, and tested positive, and no spread of infection to other employees was confirmed). The period from the first patient’s positive diagnosis (Day 1) to the end of treatment for the third patient (Day 19) lasted 19 days. One patient was asymptomatic and two had mild symptoms (according to the COVID-19 diagnostic criteria^12^). At the end of treatment, one patient still had a taste disorder, which was later confirmed to have improved. Regarding SARS-CoV-2 vaccination history, four had completed two doses and three had not been vaccinated. Of the three positive cases, one had a history of vaccination and was asymptomatic. One patient had not been vaccinated and tested negative for COVID-19; however, she adhered to infection control measures and spent her time quietly in a private room. Of the three negative patients who had a history of vaccination, one complied with infection control measures, while the others had difficulty following the measures.

**Figure 1:**
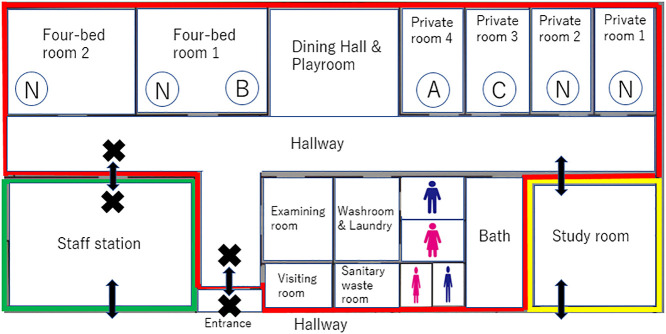
Zoning and placement of patients in the child and adolescent psychiatric ward The red zone (contaminated area), the yellow zone (intermediate area), and the green zone (clean area) are shown in their respective colors. Access to the ward through the ward entrance and staff station was prohibited. Staff entered the ward through the yellow zone after donning personal protective equipment (PPE) in a separate room not shown. Additionally, staff were only permitted to leave the ward after removing their PPE in the yellow zone. The patient placement in each ward as of Day 1 is indicated by circles. N is a COVID-19 negative patient, A, B, and C are positive patients. After Case A was transferred to the infection control ward on Day 3, Case B was transferred to private room 4 on Day 4. Case B subsequently tested positive on Day 9 and was transferred to the infection control ward along with Case C.

Based on the advice of the nosocomial infection control committee, it was difficult to isolate only COVID-19-positive patients in the ward. Therefore, zoning was performed as follows: the child and adolescent psychiatric ward was designated as a red zone (contaminated area) from Day 1 to Day 10. Following this, it was designated as a yellow zone (intermediate zone) and then as a green zone (clean zone) (Figure 1). During treatment, activities where patients gathered, such as sport time, occupational therapy, and group psychotherapy, were suspended. Patients were also instructed to maintain physical distance from others, wear a mask, eat at their bedside, and disinfect their hands. However, due to patients finding it difficult to follow infection prevention measures, there was a possibility of increasing the risk of infection spread. As such, it was decided that an isolated infection control ward, where negative pressure treatment is possible, should be set up promptly. Consequently, starting on Day 3, patients who tested positive were transferred to the infection control ward until the end of treatment. During this time, all staff wore personal protective equipment such as face shields, N95 masks, gloves, and gowns while caring for patients. Written consent for publication was obtained from each of the three cases presented below.

## Case reports

### Case A (14-year-old girl with autism spectrum disorder)

No history of SARS-CoV-2 vaccination. Day 0: an employee tested positive for COVID-19. As the patient had close contact with the employee while not wearing a mask and conversing with them, a SARS-CoV-2 nucleic acid amplification test (transcription reverse-transcription concerted reaction: TRC) was performed, which was negative. Body temperature of 37.5 degrees. Day 1: cough and nasal discharge. A body temperature of 37.9 degrees. The SARS-CoV-2 antigen qualitative test was positive (first day of COVID-19 infection). Day 2: advice on responding to COVID-19 was obtained from a pediatric infectious disease specialist from another hospital. The patient had a sore throat, a headache, and coughed. Body temperature of 40.4 degrees. A chest CT scan showed no abnormalities such as pneumonia. Day 3: a body temperature of 36.6 degrees. Blood tests showed a decrease in white blood cells to 2,500/μL, an increase in aspartate aminotransferase (AST) to 76 U/L, and alanine aminotransferase (ALT) to 81 U/L. A taste disorder appeared and the patient was transferred to the infection control ward. Day 8: cough and nasal discharge improved. Day 10: while the taste disorder persisted, the patient returned to the child and adolescent psychiatric ward and was released from the red zone on the same day. Day 11: normalization of white blood cells, AST, and ALT was confirmed. Day 15: the patient was discharged from the hospital. Improvement of the taste disorder was confirmed at the subsequent outpatient visit. Additionally, case A was often bedridden due to fever and other factors during the early stages of the illness. However, as the fever subsided, she began to spend the day at her own pace. Although the patient appeared to be restless, walking around due to having too much free time, etc., no obvious psychobehavioral problems were observed.

### Case B (14-year-old girl with post-traumatic stress disorder)

No history of SARS-CoV-2 vaccination. Day 0: an employee tested positive for COVID-19. Although the patient was asymptomatic, she had close contact with the infected employee. Consequently, a SARS-CoV-2 nucleic acid amplification test was performed, which was negative. Day 1: body temperature ranged from 37.1 to 37.5 degrees. The SARS-CoV-2 antigen qualitative test was negative. Day 2: the patient had a cough and a sore throat. However, the SARS-CoV-2 nucleic acid amplification test was negative. Day 3: case A transferred to the infection control ward. Day 4: the patient had a cough, malaise, and a body temperature of 37.3 degrees. The patient was moved from a four-bed room to a private room. Day 7: a body temperature of 37.1 degrees. The SARS-CoV-2 antigen qualitative test was negative. Day 8: a body temperature of 37.1 degrees. The patient took off her mask and had a conversation with Case C and others in the washroom. Day 9: body temperature rose to 39.3 degrees. The SARS-CoV-2 antigen qualitative test was positive, and the patient was transferred to the infection control ward (COVID-19 onset day 0). Day 10: a body temperature of 39.8 degrees. The patient had a cough and a sore throat. Day 11: body temperature was 38.3 degrees in the morning, but subsequently improved to 36.7 degrees. The patient no longer had a cough or a sore throat. A chest CT scan showed no abnormalities such as pneumonia. A blood test showed a decrease in white blood cells to 2,400/μL and an increase in lymphocytes to 51.7%. After day 16, no subjective or objective symptoms were present. Day 19: the patient returned to the child and adolescent psychiatric ward. Case B admitted to 10 days of self-injury such as wrist cutting during the 19 days immediately before the observation period. However, no self-injury was observed on Days 1–19 of the observation period. The normalization of white blood cells and lymphocytes after the end of the treatment period was confirmed.

### Case C (14-year-old girl with autism spectrum disorder)

The patient had completed two doses of SARS-CoV-2 vaccination. Day 0: an employee tested positive for COVID-19. Day 1: Case A tested positive. Although the patient was asymptomatic, a SARS-CoV-2 antigen qualitative test was performed as the patient had close contact with an infected individual. The test was negative. The patient took off her mask and played with other children. Day 3: Case A was transferred to the infection control ward. However, she was still unable to keep herself quiet in her room and hated wearing a mask. In addition, she repeatedly visited other rooms. Day 9: Case B tested positive. As the patient continued to talk with Case B without wearing a mask, they were deemed as having had close contact. After starting isolation, the patient was transferred to the infection control ward. Day 11: the SARS-CoV-2 nucleic acid amplification test was positive (COVID-19 onset day 0). However, the patient was asymptomatic. Day 19: the patient remained asymptomatic until the 8th day after onset and returned to the child psychiatric ward after completing the health observation period. There was a remarkable refusal to wear a mask due to hypersensitivity, and the patient stated that the mask sticks to her face, that it feels strange, and that it is difficult to breathe. In addition, as Case C was asymptomatic, she occasionally complained about the isolation but gradually began to spend time at her own pace with TV, comics, letters, etc. Case C presented no apparent psychobehavioral problems.

## Discussion

To our knowledge, this study is one of few reports describing a COVID-19 outbreak in a child and adolescent psychiatric ward. Therefore, it is important to consider the clinical course of COVID-19, as well as the course of psychiatric symptoms, psychiatric diagnoses, and responses based on social background from various angles.

Regarding the symptoms of COVID-19, both symptomatic patients had mild upper respiratory tract inflammation symptoms, which improved with the administration of acetaminophen and the encouragement of fluid intake. Similar to previous reports,^9–11^ no further medical intervention or specialized pediatric care was required. Changes in leukocytes, lymphocytes, AST/ALT, and taste disorders were also transient and subsequently improved.

Regarding infection control, it was difficult to isolate only the individuals who tested positive in the ward. At the time of the onset of the first case, all other patients were in close contact, and the possibility of the appearance of new positive cases was considered to be high. Hence, the entire ward was designated as a red zone, and to prevent the spread of infection, treatments were forced to be suspended where patients gathered together. Additionally, although the patients were instructed regarding various infection control measures, some found it difficult to follow the measures, such as avoiding visits to other rooms and not talking and eating at close distances without wearing a mask. In particular, patients with autism spectrum disorders struggled with wearing masks. The reasons for this may be discomfort due to sensory hypersensitivity, and difficulty in communication due to their mouths being covered, and it is difficult to read other people’s expressions and words. In addition, these patients continued to play and make noise in the hall, similar to before the outbreak. The reason for this was considered to be the difficulty of adapting, which made it challenging for patients to deal with disturbances in their hospitalization routines. As a result, these were considered to be among the factors that spread the infection. Regarding this, Tamon et al. found that during the COVID-19 pandemic, autistic children with restricted interests and repetitive behavior had more difficulty with social cognition while wearing masks,^13^ which is consistent with this study’s findings.

Tilmanne et al. reported that strict infection control measures during a COVID-19 outbreak in a child and adolescent psychiatric ward prevented the spread of infection^9^ but did not mention patient compliance behavior. Krass et al. reported that pediatric patients who tested positive for COVID-19 in the hospital were grouped in a single ward for COVID-19-positive patients. Consequently, patients were required to maintain a certain distance from staff but had no restrictions on interaction or group activities among themselves.^10^ Similarly, in Israeli et al.’s report, COVID-19-positive and negative patients were managed in separate wards, which necessitated restrictions on contact with the outside world. However, no other restrictions were mentioned, and no new infections occurred.^11^ The entire ward in this study was marked as a red zone on the day a COVID-19-positive patient emerged. However, positive and negative patients had to spend time in the same space, and it was difficult for them to comply with infection control measures, which may have spread the infection. In addition, the fact that case B was found to be positive eight days after case A was found to be positive was considered to be due to case C being an asymptomatic infected individual. Thus, considering the incubation period, it is desirable to isolate patients as soon as possible. However, securing treatment resources, such as the staff system and infection control materials, may initially be challenging.

Even among COVID-19-negative patients, the ward was designated as a red zone, and various reactions were observed due to restrictions on staff care and ward activities. Conflicts arose between patients who adhered to infection control measures and those who did not adhere to them. The former complained to the latter that “it was their fault that the infection spread,” and they displayed their anger toward the latter by acting out, kicking walls, etc. In contrast, it was observed that the latter complained to the former that they were “saying insults” and “blaming others,” complaining that the former were “noisy” when they kicked the walls. A COVID-19-negative patient, who had adhered to infection control measures, self-harmed eight times during the ten days of the red zone, compared to no attempts in the ten days immediately preceding the outbreak. In the meantime, flashbacks and similar symptoms moved into the background. In contrast, Case B, a COVID-19-positive subject, frequently admitted to self-harm before infection but did not self-harm during the infection period. Reportedly, depressive and anxiety symptoms worsen during the early stages of the disease, while externalizing behavior increases as physical health improves in COVID-19-positive individuals.^10^ However, according to another report, both positive and negative individuals do not experience psychobehavioral problems or aggravation of suicide-related symptoms.^11^ As the trend was unclear, it is necessary to accumulate relevant knowledge in the future.

Additionally, all patients had a history of child guidance center intervention. As a result, this study was unable to directly inform their families of the COVID-19 outbreak and infection. Moreover, unlike what was attempted by Israeli et al.,^11^ the majority of this study’s patients did not have the option of being discharged to home. In addition, similar to Thompson et al.’s study,^14^ various issues, including the ethical issue of how to balance infection control and behavioral restrictions such as isolation and restraint, were highlighted in this study.

A child and adolescent psychiatric ward is not only a place to improve mental symptoms but also a place to support child development and a place where they live for a relatively long period, ranging from several months to over a year. Due to the outbreak of COVID-19, the children’s daily lives were severely disrupted, and the focus of care was redirected to infection control, which placed a heavy burden on both the children and the staff.

Owing to current circumstances, with no end to the COVID-19 pandemic in sight, it is necessary to make use of this experience to minimize the disadvantages children face in the hospital environment, to prevent the onset of COVID-19, and to better respond when infection recurs.

## Supplementary Material

PDF-Japanese
