# Supplementary material for: Outbreak of novel coronavirus infection (COVID-19) in a child and adolescent psychiatric ward: Characteristics and responses
Source: Fujita Med J. 2023 Aug 28;9(4):301–5. doi: 10.20407/fmj.2023-003 (PMC10701222; doi:10.20407/fmj.2023-003)
Supplement: Supplementary file 1 — PDF-Japanese [file fmj-9-301-s001.pdf]

Title: 児童思春期精神科病棟における新型コロナウイルス感染症（COVID-19）発生－その特徴と対応－

Running head: 児童思春期精神科病棟における COVID-19 発生

Koichi Furuhashi MD, PhD[1,2]

[1]Department of Child and Adolescent Psychiatry, National Hospital Organization Higashiowari National Hospital, Nagoya, Aichi, Japan

[2]Department of Psychiatry, Fujita Health University, Toyoake, Aichi, Japan

Case report

Corresponding author: Koichi Furuhashi MD, PhD

Department of Child and Adolescent Psychiatry, National Hospital Organization Higashiowari National Hospital

2-1301 Omori-kita, Moriyama-ku, Nagoya, Aichi 463-0802, JAPAN

Phone：+81-52-798-9711

E-mail: fkoichi@med.nagoya-u.ac.jp

抄録

背景：新型コロナウイルス感染症（COVID-19）は、未だ終息の見通しが立っていない。現在、行動制限を緩和し、社会経済活動の維持を図る政策が強調される一方、精神科病院での感染管理の困難さが問題となっている。児童精神科領域では、メンタルヘルスや、外来・入院治療環境への影響が報告されてきた。私たちは児童思春期精神科病棟において COVID-19 の発生を経験したが、同様の報告は少なく、経験の蓄積は意義あると考えた。

症例提示：7 名が入院する児童思春期精神科病棟で発生したクラスターで、全て 14 歳女児である 3 人の COVID-19 陽性が確認された。2 人は上気道炎症状に留まり、1 人は無症状だった。精神科主診断は、1 人は心的外傷後ストレス障害、2 人は自閉症スペクトラム障害だった。病棟全体をレッドゾーン（汚染区域）として、集団活動の中止やマスク着用、患者間の距離の確保など感染対策を講じたが、患者の遵守は困難であり、感染管理病棟の利用も必要とした。

結論：児童思春期精神科病棟での COVID-19 クラスターにおける感染管理は、患児の特性や症状の影響もあり、困難であり、陽性・陰性問わず、活動やケアが制限されることによる精神・行動上の影響もあった。感染管理とよりよい治療環境の両立を図るべく、今回の経験を活かし入念な準備が必要と考えた。

キーワード

新型コロナウイルス感染症（COVID-19）、 児童思春期精神科病棟、入院患者、心的外傷後ストレス障害、自閉症スペクトラム障害

序論

新型コロナウイルス感染症（COVID-19）は、2019 年末から急速に全世界へ広がり、3 年を経過してもなお新たな変異株の出現による感染の波が続いており、終息の見通しが立っていない。そうした中、行動制限を緩和しながら、社会経済活動の維持を図っていく政策が提唱されている。しかし、医療機関では一度感染が広がると、通常の医療の維持が困難となり、特に精神科病院での対応の困難さは、すでに多くの報告がある。精神障害者は、肥満、高血圧、メタボリックシンドロームなどの身体的併存疾患の割合が高く、COVID-19 に感染しやすく、また重症化しやすい可能性が指摘されている[1]。入院環境では、閉鎖病棟も存在し、病棟内でのソーシャルディスタンスの確保、消毒剤設置（誤飲の発生リスク）、マスク装着等の衛生管理の徹底が難しい。またスタッフ教育、スキルの不十分さや、組織や地域社会のサポートが脆弱であることなども指摘されている[1,2]。COVID-19 の治療についても、精神科病院内での医療資源は限界があり、精神疾患をもつ感染者の治療を引き受ける総合病院も受け入れが困難であることが指摘されている[3]。これらのことから、引き続き厳重な感染対策が求められている。

児童精神科領域では、子どものメンタルヘルスへの影響、外来・入院などの治療環境への影響などが報告されてきた。例えば、不安や抑うつ症状の増加、希死念慮、自傷、自殺の増加、さらに神経発達症などの神経多様性のある子どもは、より高い心理的苦痛や行動上の問題が大きいことなどが指摘された[4,5]。臨床への影響としては、入院・外来患者数の減少や、入院患者の外出・外泊の制限、集団活動の中止、入院中の行動制限により、衝動性や攻撃性の適応的な発散が困難となり、病棟内でのトラブル増加、相対的な入院患者の重症度の上昇、入院期間の長期化などが指摘された[6]。また、児童思春期精神科病棟への緊急入院患者数の増加、そのうち女子の割合や自殺傾向の増加の報告や[7]、児童思春期精神科病棟に入院した過半数の患者が、COVID-19 に関連するストレッサーが要因となっていたとの報告[8]などがあげられる。

今回私たちは、児童思春期精神科病棟で複数の患者に COVID-19 の発症を経験した。当院では、入院患者の感染は初めての経験であり、感染対策委員会での助言を得て、慎重に対応した。Tilmanne らは、児童思春期精神科病棟内で COVID-19 発生について、厳格な感染対策により感染拡大を制御できた事例を報告した[9]。また、Krass らは、精神科病院での COVID-19 の発生について、陽性の児童思春期患者は小児 COVID-19 ユニットに集められ、吐き気・嘔吐、咳、咽頭痛、息切れといった症状を 20〜30%台に認めたが、酸素投与や治療目的の転院を要するものはいなかったこと、病初期から抑うつや不安症状が高まったことを報告した[10]。さらに、Israeli らは、児童思春期精神科病棟での COVID-19 の発生により、面会や外出・外泊の制限などの外部との接触の制限のため、精神科治療にも影響を及ぼしたこと、陽性者のほとんどは軽症または無症状であったこと、陽性者と陰性者でユニットを分けたが、主にスタッフ側の感染対策で困難を生じたこと、それにも関わらず、患者の臨床状態は安定しており、精神行動上の問題、自殺関連症状の悪化は認めなかったことを報告した[11]。

COVID-19 は新規感染症であり、病態の解明や治療法の開発は日々発展中であることや、各国・地域による COVID-19 に対する社会的・法的規制、児童思春期精神科の治療資源の違いがあることや、前述の通り、児童思春期精神科病棟での COVID-19 発症対応の報告は、これまで数例に留まっており、さらなる経験の蓄積が必要と考えた。それゆえ、私たちの経験の報告は意義あること考え、以下に示すものである。

クラスターの経過と感染対策

当院は精神科専門病院であり、内児童思春期精神科病棟は 14 床を占め、構成は個室 4 部屋、4 人室 2 部屋、保護室 2 部屋である。2022 年 4 月から 5 月にかけての観察期間中に 7 人の患者（年齢 13〜15 歳、全員女児）が入院していた。精神科主診断は、心的外傷後ストレス障害が 5 名、自閉症スペクトラム障害が 2 名だった。養育者からの虐待などを理由に、

全員が児童相談所の介入歴があった。観察期間中 3 名の患者が COVID-19 陽性と判明した。感染源は職員（看護師）と推定された。以下、職員の陽性判明日を Day0 とした（自宅で発熱したため近医を受診し陽性が判明し、他の職員への感染の波及は認めなかった）。患者 1 例目の陽性判明（Day1）から、3 例目の療養終了（Day19）までに 19 日を要した。1 名は無症状、2 名は軽症だった（COVID-19 診断基準 [12] による）。療養終了時に 1 名に味覚障害が残存したが、その後改善を確認した。SARS-CoV-2 ワクチン接種歴は、4 人が 2 回接種を完了、3 人は未接種だった。陽性者 3 名中、接種歴がある者は 1 人で、無症状だった。未接種陰性者は 1 名だったが、感染対策を遵守し、個室で静かに過ごしていた。接種歴のある陰性者 3 名のうち 1 名は感染対策を遵守していたが、 2 名は感染対策を守ることが難しかった。

院内感染対策委員会の助言に基づき、病棟内で陽性者のみを隔離することは困難であったため、児童思春期精神科病棟を Day1 から Day10 までレッドゾーン（汚染区域）とし、イエローゾーン（中間区域）、グリーンゾーン（清潔区域）とゾーニングを行った（図 1）。スポーツタイム、作業療法、集団心理療法など、患者が集まって行う治療を中止とした。また患者には、他者と物理的な距離を取ること、マスクを着用すること、食事は各ベッドサイドで摂ること、手指の消毒の励行などの指導を行った。しかしながら、患者が感染予防対策を守ることが困難で、感染拡大リスクを高める可能性があったため、閉鎖していた陰圧管理のできる感染管理病棟を急遽立ち上げることとし、Day3 以降、陽性者を療養終了まで感染管理病棟に転棟させた。この間、全ての職員は、フェイスシールド、N95 マスク、手袋、ガウンなどの個人用保護具（Personal Protective Equipment：PPE）を装着し、患者のケアにあたった。尚、以下に提示する 3 人の症例からはいずれも書面にて論文掲載への同意を取得した。

症例報告

症例 A（14 歳女児、自閉症スペクトラム障害）

SARS-CoV-2 ワクチン接種歴なし。Day0：職員の COVID-19 陽性が判明。その職員とマスクを着用せず会話した濃厚接触者であったため、SARS-CoV-2 核酸増幅検査（Transcription Reverse-transcription Concerted reaction：TRC）を実施し陰性。体温 37.5 度。Day1：咳、鼻汁あり。体温 37.9 度。SARS-CoV-2 抗原定性検査は陽性（COVID-19 発症 1 日目）。Day2：他院の小児感染症専門医から COVID-19 対応に関する助言を得た。咽頭痛、頭痛、咳あり。体温 40.4 度。胸部 CT では、肺炎像など異常所見を認めず。Day3：体温 36.6 度。血液検査で、白血球 2,500/μL と低下、aspartate aminotransferase（AST）76U/L、alanine aminotransferase（ALT）81U/L と増加。味覚障害出現。感染管理病棟に転棟。Day8：咳、鼻汁は改善。Day10：味覚障害継続、児童思春期精神科病棟に戻った。同日レッドゾーンを解除。Day11：白血球と AST、ALT の正常化を確認。Day15：退院。その

後の外来通院時に味覚障害の改善を確認した。また症例 A は、病初期は発熱などのため臥床傾向にあったが、解熱するに従って、暇を持て余し、歩き回るなどの落ち着かない様子もありつつ、次第に自分のペースで一日を過ごせるようになり、明らかな精神行動上の問題を認めなかった。

症例 B（14 歳女児、心的外傷後ストレス障害）

SARS-CoV-2 ワクチン接種歴なし。Day0：職員の COVID-19 陽性が判明。無症状だが、濃厚接触者であるため、SARS-CoV-2 核酸増幅検査を実施し陰性。Day1：体温 37.1～37.5 度で経過。SARS-CoV-2 抗原定性検査は陰性。Day2：咳、咽頭痛あり、SARS-CoV-2 核酸増幅検査は陰性。Day3：症例 A が感染管理病棟に転棟。Day4：咳、倦怠感あり。体温 37.3 度。大部屋から個室へ移動。Day7：体温 37.1 度。SARS-CoV-2 抗原定性検査は陰性。Day8：体温 37.1 度。マスクを外して、症例 C らと洗面所で会話をしていた。Day9：体温 39.3 度へ上昇。SARS-CoV-2 抗原定性検査が陽性となり、感染管理病棟に転棟（COVID-19 発症 0 日目）。Day10：体温 39.8 度。咳、咽頭痛あり。Day11：朝の体温 38.3 度だったが、その後 36.7 度へ改善。咳、咽頭痛なし。胸部 CT では、肺炎像など異常所見を認めず。血液検査で、白血球 2,400/µL と低下、リンパ球 51.7%と増加。Day16 以降、自覚・他覚症状なし。Day19：児童思春期精神科病棟に戻った。また症例 B は観察期間の直前 19 日間でリストカットなどの自傷行為をしていた日が 10 日あったが、観察期間の Day1-19 では自傷行為を認めなかった。療養期間終了後の白血球、リンパ球の正常化を確認した。

症例 C（14 歳女児、自閉症スペクトラム障害）

SARS-CoV-2 ワクチン接種 2 回済。Day0：職員の COVID-19 陽性が判明。Day1：症例 A が陽性判明。無症状だが、濃厚接触者につき、SARS-CoV-2 抗原定性検査を実施し陰性。マスクを外して他児と騒いでいた。Day3：症例 A が感染管理病棟に転棟した。その後も自室での安静を守れず、マスク着用を嫌がった。さらに、他室への訪問を繰り返した。Day9：症例 B の陽性が判明。症例 B とマスク着用せず会話を続けていたことから、濃厚接触者となった。隔離開始した上で、感染管理病棟に転棟。Day11：SARS-CoV-2 核酸増幅検査で陽性（COVID-19 発症 0 日目）。無症状。Day19：発症 8 日目まで無症状継続し、健康観察期間終了したため、児童精神科病棟に戻った。マスクは顔に張り付くからいや、へんな感じがする、息苦しいと、感覚過敏によるマスク拒否が顕著だった。また症例 C は、無症状であることから、隔離の不満を訴えることはあったが、次第に自分のペースでテレビや漫画、手紙などで時間を過ごすようになり、明らかな精神行動上の問題を認めなかった。

考察

私たちの知る限り、これは児童思春期精神科病棟における COVID-19 発生に関する数少ない報告の一つになる。そのため COVID-19 の臨床経過はもとより、精神症状の経過、精

神科的診断や社会的背景を踏まえた対応について様々な角度から検討することは重要と考えた。

まず、COVID-19 の症状については、有症状の 2 名とも軽度の上気道炎症状が中心で、アセトアミノフェンの投与や、水分摂取の励行でほどなく改善し、それ以上の医学的介入、小児科での専門治療が不要であったことは、これまでの報告[9-11]と同様であった。白血球やリンパ球、AST/ALT の変化や味覚障害についても、一過性のものでその後改善を認めた。

感染管理については、病棟内で陽性者のみを隔離することが困難であったこと、一例目の発症時、他の患者は全て濃厚接触者であり、新たな陽性者の出現の可能性が高いと思われたことから、病棟全体をレッドゾーンとし、感染拡大防止のため、患者が集まって行う治療を中止とせざるを得なかった。また患者には、様々な感染対策の指導を行ったが、一部の患者が他室へ訪問したり、マスク着用をせず近い距離で会話や食事をするなど、感染対策を守ることが困難であった。特に、自閉症スペクトラム障害の患者はマスク着用が苦手であったが、その理由として、感覚過敏性のため不快感を覚えたり、口元を覆い他者の表情やことばを読み取りにくいため、コミュニケーション上の困難さを感じることが考えられた。また、発生前のように、ホールで遊んだり、騒いだりすることを続けていたが、その理由として、柔軟な適応が困難であるため、入院生活のルーチンが崩れることのとまどいがあったことが考えられた。結果としてこれらが感染を広げる要因の一つとして考えられた。これに関しては、Tamon らが、COVID-19 パンデミック下で、興味の限局と反復行動（Restricted Interests and Repetitive Behavior：RRB）を有する自閉症児が、マスクを着用中に社会的認知がより困難となることを報告しており[13]、私たちの経験とも一致する。

Tilmanne らは、児童思春期精神科病棟で COVID-19 が発生した際の厳格な感染予防対策により、感染拡大を防止したと報告したが[9]、患者の遵守行動についての言及はなかった。Krass らの報告では、院内で COVID-19 陽性となった小児患者を、一か所の COVID-19 陽性者用の病棟に集めたため、スタッフとは一定の距離をとる必要があったが、患者間での交流やグループ活動は制限されなかった[10]。同様に Israeli らの報告でも、COVID-19 陽性者・陰性者で病棟を分けて管理したため、外部との接触に関しての制限はあったが、それ以外の制限について言及はなく、新たな感染例は発生しなかった[11]。私たちは、COVID-19 陽性患者が発生した日に病棟全体をレッドゾーンとしたが、陽性者及び陰性者が同じ空間で過ごすこととなり、かつ患者の感染対策遵守が困難であったこともあり、感染が広がった可能性があると考えた。また、症例 B の陽性判明が、症例 A の陽性判明の 8 日後となったことは、症例 C が無症状感染者であったことも要因として考えられた。このように、潜伏期間も考慮すると、できるだけ早く分離することが望ましいが、スタッフの体制や感染対策資材などの治療資源の確保が課題となると考えた。

COVID-19 陰性者でも、病棟がレッドゾーンとなり、スタッフによるケアや病棟活動が制約されたことによる様々な反応が見られた。感染対策を遵守している患者と、守ることが苦手な患者の間で軋轢が生じた。前者は後者に対して、“あいつらのせいで感染が広がった”と訴えたり、後者に対する怒りを、壁を蹴るなどの行動化で示した。一方後者は前者に対して、“悪口をいってくる”“人のせいにしてくる”と訴えたり、前者が壁を蹴る音に対して「うるさい」と訴えるなどの場面が観察された。ある陰性患者は、感染対策を遵守していたが、発生直前の 10 日間では 1 回もなかった自傷行為が、レッドゾーンの 10 日間で 8 回認めたが、その間フラッシュバックなどは後景化した。一方で陽性者である症例 B は感染前に頻繁に自傷行為を認めたが、感染期間中は 1 回もなかった。陽性者において、病初期から抑うつや不安症状が悪化し、身体的健康が回復するにつれて、外在化行動が増加したり [10]、陽性者・陰性者問わず、精神行動上の問題、自殺関連症状の悪化は認めなかった [11] 等、様々な報告があり、一定の傾向は明確でなかったことから、今後も様々な知見を集積する必要があると考えた。

また全員が児童相談所の介入歴があるため、COVID-19 発生や感染の事実を、家族に直接伝えることができなかったり、Israeli らが試みたように [11]、家庭に退院させるという選択肢がない患者が大半を占めた。また、Thompson らの指摘の通り [14]、感染対策と隔離や拘束などの行動制限のバランスをいかにとるべきかという倫理的問題も含め、様々な課題を浮き彫りにした。

児童思春期精神科病棟は、精神症状を改善させるだけに留まらず、子どもの発達を支援する場であり、数か月〜長いと 1 年を超える比較的長期間の生活を送る場でもある。COVID-19 発生ため、子どもの日常が失われ、感染管理中心のケアにならざるを得なかったことは、子どもにも、職員にも大きな負担となった。

COVID-19 終息の見通しが立たない状況下で、入院環境下での子どもの不利益を最小限にし、COVID-19 の発症予防と再発時のよりよい対応のために、今回の経験を十分に活かすことが求められている。

謝辞
本報告は、いかなる研究費の支援を受けなかった。本論文の作成にあたり、感染症の専門医の観点から助言をいただいた、藤田医科大学医学部感染症科、土井洋平教授に深く感謝申し上げる。また、患者の協力に深く感謝申し上げる。

利益相反

著者は本報告に関する利益相反はない。

引用文献

1. Houben F, van Hensbergen M, den Heijer CDJ, Dukers-Muijrers NHTM, Hoebe CJPA. Correction to: Barriers and facilitators to infection prevention and control in Dutch psychiatric institutions: a theory-informed qualitative study. BMC Infect Dis 2022;22:394.
2. Zoghbi M, Haddad C, Khansa W, Karam E, Chamoun A, Hachem D. COVID-19 outbreak in a psychiatric hospital: what makes it worse? Ann Gen Psychiatry 2022;21:26.
3. Usuda K, Okazaki E, Tsukie Y, Yamanouchi Y. The impact of COVID-19 on inpatient wards at psychiatric hospitals in Japan. Asian J Psychiatr 2021;62:102720.
4. Pustake M, Mane S, Ganiyani MA, Mukherjee S, Sayed M, Mithbavkar V, Memon Z, Momin AS, Deshmukh K, Chordia A, Parida S, Johnson A, Warghade S, Varma D, Bhagwat A. Have the COVID-19 pandemic and lockdown affected children's mental health in the long term? A repeated cross-sectional study. BMJ Open 2022;12:e058609.
5. Samji H, Wu J, Ladak A, Vossen C, Stewart E, Dove N, Long D, Snell G. Review: Mental health impacts of the COVID-19 pandemic on children and youth - a systematic review. Child Adolesc Ment Health 2022;27:173-89.
6. Shibata S. The impact from COVID-19 pandemic on the practice of child and adolescent psychiatry. Japanese Journal of Clinical Psychiatry 2020;49:1525-9 (in Japanese).
7. Sevecke K, Wenter A, Schickl M, Kranz M, Krstic N, Fuchs M. Inpatient care capacities in child and adolescent psychiatry-increase in emergency admissions during the COVID-19 pandemic?. Neuropsychiatr 2023;37:12-21.
8. Reece L, Sams DP. The impact of COVID-19 on adolescent psychiatric inpatient admissions. Clin Child Psychol Psychiatry 2022;27:112-21.
9. Tilmanne A, De Crombrugghe G, Al-Husni Al-Keilani M, Le Loc'h G, Delvenne V, Smeesters PR. A well-controlled Covid-19 cluster in a semi-closed adolescent psychiatry inpatient facility. Clin Microbiol Infect 2021;27:153-4.
10. Krass P, Zimbrick-Rogers C, Iheagwara C, Ford CA, Calderoni M. COVID-19 Outbreak Among Adolescents at an Inpatient Behavioral Health Hospital. J Adolesc Health 2020;67:612-4.
11. Israeli S, Kagan I, Yerushalmi S, Gelman S, Yarkoni I, Levitan L, Argo D, Kohn Y. COVID-19 outbreak in a child and adolescent psychiatric ward: Challenges and lessons to be learned -Case study. J Child Adolesc Psychiatr Nurs 2023;36:17-20.
12. Ministry of Health, Labour and Welfare. Shingata korona uirusu kansensho (covid-

19) shinryo no tebiki (Handbook for COVID-19 Treatment for Novel Coronavirus Infection). Version 9.0 (in Japanese) <https://www.mhlw.go.jp/content/000936655.pdf> (Accessed March 19, 2023)

13. Tamon H, Itahashi T, Yamaguchi S, Tachibana Y, Fujino J, Igarashi M, Kawashima M, Takahashi R, Shinohara NA, Noda Y, Nakajima S, Hirota T, Aoki YY. Autistic children and adolescents with frequent restricted interest and repetitive behavior showed more difficulty in social cognition during mask-wearing during the COVID-19 pandemic: a multisite survey. BMC Psychiatry 2022;22:608.

14. Thompson AD, Berkman ER, Simmons SW, Porter KM, Kroon L, Goldman R, Ramasamy RS. Ethical Considerations in Balancing Use of Seclusion and Restraint With Risk of COVID-19 Exposure: Recommendations for Youth Inpatient Psychiatry Units. J Am Acad Child Adolesc Psychiatry 2022;61:1319-21.

図１　児童思春期精神科病棟のゾーニングと患者配置

赤枠で囲まれたレッドゾーン（汚染区域)、黄枠で囲まれたイエローゾーン（中間区域)、緑枠で囲まれたグリーンゾーン（清潔区域）をそれぞれ示した。病棟入り口、スタッフステーションから病棟への出入りは禁止された。スタッフは、図に示されていない別室で個人用保護具（PPE）を着用後、イエローゾーンを通じて病棟に入る。また、病棟から出る際は、イエローゾーンで PPE を脱衣後、外に出る。

また、各病室の Day1 時点での患者配置を丸印で示した。N が COVID-19 陰性患者、A・B・C は各陽性患者である。Day3 で症例 A が感染管理病棟へ転棟後、Day4 で症例 B が個室 4 へ転室した。その後症例 B は、Day9 で陽性となり、症例 C とともに感染管理病棟へ転棟した。

Figure1

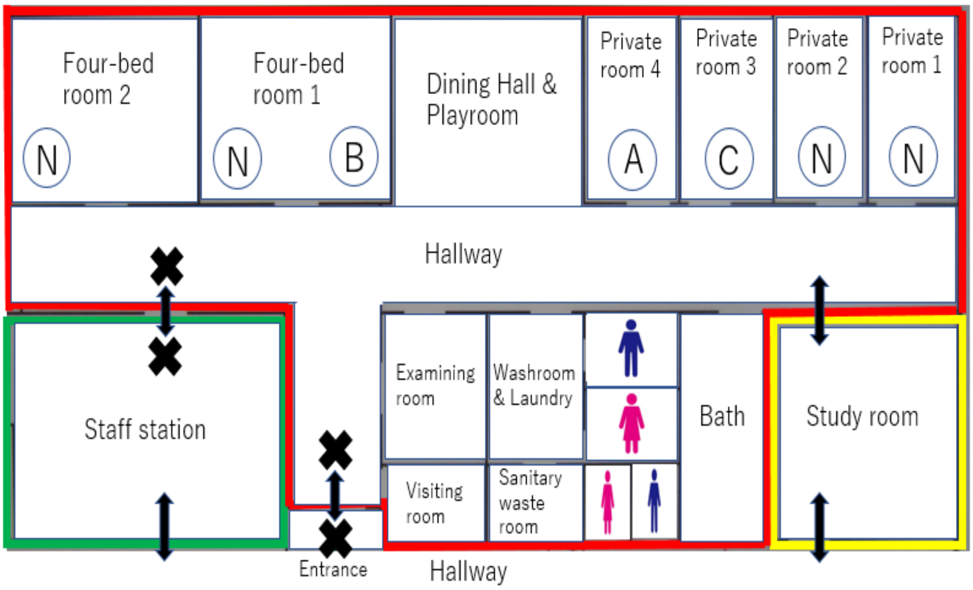
Four-bed room 2
N
Four-bed room 1
N
B
Dining Hall & Playroom
Private room 4
A
Private room 3
C
Private room 2
N
Private room 1
N
Hallway
Staff station
Examining room
Washroom & Laundry
Visiting room
Sanitary waste room
Bath
Study room
Entrance
Hallway
